# Safety Monitoring of Bivalent COVID-19 mRNA Vaccine Booster Doses Among Persons Aged ≥12 Years — United States, August 31–October 23, 2022

**DOI:** 10.15585/mmwr.mm7144a3

**Published:** 2022-11-04

**Authors:** Anne M. Hause, Paige Marquez, Bicheng Zhang, Tanya R. Myers, Julianne Gee, John R. Su, Phillip G. Blanc, Alisha Thomas, Deborah Thompson, Tom T. Shimabukuro, David K. Shay

**Affiliations:** ^1^CDC COVID-19 Emergency Response Team; ^2^Food and Drug Administration, Silver Spring, Maryland.

On August 31, 2022, the Food and Drug Administration (FDA) authorized bivalent formulations of BNT162b2 (Pfizer-BioNTech) and mRNA-1273 (Moderna) COVID-19 vaccines; these vaccines include mRNA encoding the spike protein from the original (ancestral) strain of SARS-CoV-2 (the virus that causes COVID-19) and from the B.1.1.529 (Omicron) variants BA.4 and BA.5 (BA.4/BA.5). These bivalent mRNA vaccines were authorized for use as a single booster dose ≥2 months after completion of primary series or monovalent booster vaccination; Pfizer-BioNTech bivalent booster was authorized for persons aged ≥12 years and Moderna for adults aged ≥18 years.*^,^^†^ On September 1, 2022, the Advisory Committee on Immunization Practices (ACIP) recommended that all persons aged ≥12 years receive an age-appropriate bivalent mRNA booster dose.^§^ To characterize the safety of bivalent mRNA booster doses, CDC reviewed adverse events and health impacts reported after receipt of bivalent Pfizer-BioNTech and Moderna booster doses during August 31–October 23, 2022, to v-safe,^¶^ a voluntary smartphone-based U.S. safety surveillance system established by CDC to monitor adverse events after COVID-19 vaccination, and the Vaccine Adverse Event Reporting System (VAERS),** a U.S. passive vaccine safety surveillance system managed by CDC and FDA (*1*). During August 31–October 23, 2022, approximately 14.4 million persons aged ≥12 years received a bivalent Pfizer-BioNTech booster dose, and 8.2 million adults aged ≥18 years received a bivalent Moderna booster dose.^††^ Among the 211,959 registrants aged ≥12 years who reported receiving a bivalent booster dose to v-safe, injection site and systemic reactions were frequently reported in the week after vaccination (60.8% and 54.8%, respectively); fewer than 1% of v-safe registrants reported receiving medical care. VAERS received 5,542 reports of adverse events after bivalent booster vaccination among persons aged ≥12 years; 95.5% of reports were nonserious and 4.5% were serious events. Health care providers and patients can be reassured that adverse events reported after a bivalent booster dose are consistent with those reported after monovalent doses. Health impacts after COVID-19 vaccination are less frequent and less severe than those associated with COVID-19 illness (*2*).

The v-safe system allows existing registrants to report receipt of a COVID-19 booster dose and new registrants to enter information about all doses received; registrants can also indicate whether any other vaccines were administered during the same visit. On September 2, 2022, v-safe was modified to allow participants to enter up to 6 doses of a COVID-19 vaccine. Health surveys sent daily during the first week after administration of each dose include questions about local injection site and systemic reactions and health impacts experienced; registrants can provide additional information about these reactions or health impacts via free text message.^§§^CDC’s v-safe call center staff members contact registrants who indicate that medical care was received after vaccination to request more information; registrants are also encouraged to complete a VAERS report, if indicated.

VAERS accepts reports of postvaccination adverse events from health care providers, vaccine manufacturers, and members of the public.^¶¶^ Signs and symptoms and diagnostic findings in VAERS reports are assigned Medical Dictionary for Regulatory Activities preferred terms (MedDRA PTs) by VAERS staff members.*** Reports of serious events to VAERS during August 31–October 23, 2022, were reviewed by CDC physicians to form a consensus clinical impression based on available data.^†††^ Death certificates and autopsy reports were requested for any report of death. CDC physicians reviewed all available information for each decedent to form an impression about the cause of death. Using selected MedDRA PTs, a search was performed to identify possible cases of myocarditis, a rare adverse event that has been associated with mRNA COVID-19 vaccines (*2*).

A bivalent booster dose in v-safe was defined as an age-appropriate mRNA vaccine dose administered on or after August 31, 2022, for registrants who had completed at least a primary series (2 doses of Pfizer-BioNTech, Moderna, or Novavax COVID-19 vaccine or 1 dose of Janssen [Johnson & Johnson] vaccine). Local and systemic reactions and health impacts reported during the week after a bivalent booster dose vaccination were described for v-safe registrants aged ≥12 years who received a bivalent booster dose during August 31–October 23, 2022. VAERS adverse event reports after a bivalent booster dose were described by serious and nonserious classification, demographic characteristics, and MedDRA PTs. All analyses were conducted using SAS software (version 9.4; SAS Institute). These surveillance activities were reviewed by CDC and conducted consistent with applicable federal law and CDC policy.^§§§^

## Review of v-safe Data

During August 31–October 23, 2022, a total of 211,959 v-safe registrants aged ≥12 years reported receiving an age-appropriate bivalent booster dose (Table 1); 1,464 (0.7%) were aged 12–17 years, 68,592 (32.4%) were aged 18–49 years, 59,209 (27.9%) were aged 50–64 years, and 82.694 (39.0%) were aged ≥65 years. Most registrants indicated that a bivalent booster dose was their fourth (96,241; 45.4%) or fifth (106,423; 50.2%) COVID-19 vaccine dose; 122,953 (58.0%) received a Pfizer-BioNTech bivalent booster dose and 89,065 (42.0%) received a Moderna bivalent booster dose. More than one third (84,450; 39.8%) of registrants reported receiving at least one other vaccination at the same visit as bivalent booster vaccination; 83,005 (98.3%) received influenza vaccine.

**TABLE 1:** Demographic and vaccination characteristics of persons aged ≥12 years* who reported receipt of a bivalent Pfizer-BioNTech or Moderna COVID-19 vaccine booster dose to v-safe^†^ — United States, August 31–October 23, 2022

Characteristic	Vaccine, no. (%)		
	Pfizer-BioNTech n = 122,953	Moderna n = 89,006	Total N = 211,959
**Sex**			
Female	77,913 (63.4)	56,651 (63.7)	**134,564 (63.5)**
Male	44,031 (35.8)	31,697 (35.6)	**75,728 (35.7)**
Unknown	1,009 (0.8)	658 (0.7)	**1,667 (0.8)**
**Age group, yrs**			
12–17	1,464 (1.2)	NA	**1,464 (0.7)**
18–49	41,022 (33.4)	27,570 (31.0)	**68,592 (32.4)**
50–64	34,947 (28.4)	24,262 (27.3)	**59,209 (27.9)**
≥65	45,520 (37.0)	37,174 (41.8)	**82,694 (39.0)**
**Ethnicity**			
Hispanic	6,967 (5.7)	4,765 (5.4)	**11,732 (5.5)**
Non-Hispanic	112,895 (91.8)	82,009 (92.1)	**194,904 (92.0)**
Unknown	3,091 (2.5)	2,232 (2.5)	**5,323 (2.5)**
**Race**			
American Indian or Alaska Native	441 (0.4)	328 (0.4)	**769 (0.4)**
Asian	6,884 (5.6)	4,750 (5.3)	**11,634 (5.5)**
Black or African American	6,574 (5.4)	4,583 (5.2)	**11,157 (5.3)**
Native Hawaiian or other Pacific Islander	241 (0.2)	145 (0.2)	**386 (0.2)**
White	102,535 (83.4)	74,984 (84.3)	**177,519 (83.8)**
Multiracial	2,518 (2.1)	1,667 (1.9)	**4,185 (2.0)**
Other	1,873 (1.5)	1,262 (1.4)	**3,135 (1.5)**
Unknown	1,887 (1.5)	1,287 (1.5)	**3,174 (1.5)**
**Total no. of COVID-19 vaccine doses received**			
2	86 (0.1)	52 (0.1)	**138 (0.1)**
3	4,919 (4.0)	3,186 (3.6)	**8,105 (3.8)**
4	57,603 (46.9)	38,638 (43.4)	**96,241 (45.4)**
5	59,807 (48.6)	46,616 (52.4)	**106,423 (50.2)**
6	538 (0.4)	514 (0.6)	**1,052 (0.5)**
**Vaccine co-administration** ^§^			
Yes	51,713 (42.1)	32,737 (36.8)	**84,450 (39.8)**
No	71,240 (57.9)	56,269 (63.2)	**127,509 (60.2)**

**Abbreviation**: NA = not applicable.

* On August 31, 2022, the Food and Drug Administration authorized bivalent formulations of Moderna and Pfizer-BioNTech COVID-19 vaccines for use as a single booster dose ≥2 months after completing primary or booster vaccination, Pfizer-BioNTech for persons aged ≥12 years and Moderna for persons aged ≥18 years. In v-safe, a bivalent booster dose was defined as an age-appropriate mRNA dose administered on or after August 31, 2022, for registrants who completed a primary series (2 doses of Pfizer-BioNTech, Moderna, or Novavax COVID-19 vaccine or 1 dose of Janssen).

^†^ Includes registrants who completed at least one survey during days 0–7 postvaccination.

^§^ Other vaccines administered during the same visit.

In the week after receipt of the bivalent booster dose, frequency of reporting of local injection site reactions ranged from 49.7% among adults aged ≥65 years to 72.9% among adults aged 18–49 years; the prevalence of reported systemic reactions ranged from 43.5% among adults aged ≥65 years to 67.9% among adults aged 18–49 years (Table 2). The most frequently reported reactions among these age groups after bivalent booster dose vaccination were injection site pain (range = 45.0%–70.5%), fatigue (30.0%–53.1%), headache (19.7%–42.8%), myalgia (20.3%–41.3%), and fever (10.2%–26.3%).

**TABLE 2:** Adverse reactions and health impacts reported to v-safe for persons aged ≥12 years* who received a bivalent Pfizer-BioNTech or Moderna COVID-19 vaccine booster dose — United States, August 31–October 23, 2022

Event	% Reporting reaction/health impact after vaccination, by age group, yrs^†^				
	12–17 n = 1,464	18–49 n = 68,592	50–64 n = 59,209	≥65 n = 82,694	Total N = 211,959
**Any injection site reaction**	**68.7**	**72.9**	**62.0**	**49.7**	**60.8**
Itching	4.6	8.9	7.8	6.9	**7.8**
Pain	66.9	70.5	58.8	45.0	**57.3**
Redness	8.5	10.8	9.1	7.6	**9.1**
Swelling or hardness	13.7	18.4	14.7	9.9	**14.0**
**Any systemic reaction**	**59.8**	**67.9**	**55.2**	**43.5**	**54.8**
Abdominal pain	6.4	5.5	3.6	2.1	**3.6**
Myalgia	33.6	41.3	29.0	20.3	**29.6**
Chills	19.6	20.6	13.7	9.1	**14.2**
Fatigue	45.2	53.1	40.0	30.0	**40.4**
Fever	26.3	23.7	16.6	10.2	**16.4**
Headache	36.3	42.8	31.5	19.7	**30.6**
Joint pain	14.5	21.7	16.8	11.1	**16.1**
Nausea	12.4	12.9	7.9	4.5	**8.2**
Diarrhea	3.0	6.7	5.4	3.8	**5.2**
Rash	1.4	1.3	1.1	0.9	**1.1**
Vomiting	2.5	1.2	0.6	0.4	**0.7**
**Any health impact**	**26.8**	**24.2**	**17.3**	**11.6**	**17.3**
Unable to perform normal daily activities	18.4	19.8	14.7	10.6	**14.8**
Unable to attend school or work	15.6	11.3	6.0	1.6	**6.1**
Needed medical care	1.2	0.9	0.7	0.8	**0.8**
Telehealth	0.2	0.3	0.2	0.2	**0.3**
Clinic	0.8	0.4	0.3	0.3	**0.3**
Emergency visit	0.1	0.1	0.1	0.1	**0.1**
Hospitalization	0	0	0	0	**0**

* On August 31, 2022, the Food and Drug Administration authorized bivalent formulations of Moderna and Pfizer-BioNTech COVID-19 vaccines for use as a single booster dose ≥2 months after completing primary or booster vaccination, Pfizer-BioNTech for persons aged ≥12 years and Moderna for adults aged ≥18 years. In v-safe, a bivalent booster was defined as an age-appropriate mRNA dose administered on or after August 31, 2022, for registrants who completed a primary series (2 doses of Pfizer-BioNTech, Moderna, or Novavax COVID-19 vaccine or 1 dose of Janssen).

^†^ Percentage of registrants who reported a reaction or health impact at least once during days 0–7 postvaccination.

Reported inability to complete normal daily activities ranged from 10.6% among adults aged ≥65 years to 19.8% among adults aged 18–49 years. Receipt of medical care was reported by 0.8% of registrants; most received care via telehealth (0.3%) or clinic (0.3%) appointment. Hospitalization was reported by 55 (0.03%) registrants. Among 45 registrants with information about the hospitalization available from the v-safe call center or free text message response, 29 indicated that the hospitalization was unrelated to vaccination, 13 completed a VAERS report, and three did not wish to complete a VAERS report.

## Review of VAERS Data

During August 31–October 23, 2022, VAERS received and processed 5,542 reports of adverse events among persons aged ≥12 years who reported receiving a bivalent booster dose (Table 3).^¶¶¶^ The median recipient age was 60 years (range = 12–101) and 3,559 (64.2%) were female; 939 (16.9%) reports indicated at least one other vaccine was received at the same visit as booster vaccination, of which influenza vaccine was most commonly co-administered (852; 90.7%).

**TABLE 3:** Events* reported to the Vaccine Adverse Event Reporting System for persons aged ≥12 years† after receipt of a bivalent Pfizer-BioNTech or Moderna COVID-19 vaccine booster dose — United States, August 31–October 23, 2022

Adverse events	Vaccine, no. reporting (%)		
	Pfizer-BioNTech	Moderna	Total^§^
**Total**	**2,928**	**2,615**	**5,542**
**Vaccination errors^¶^**	**877 (30.0)**	**1,037 (39.7)**	**1,913 (34.5)**
Error without adverse health event	717 (81.8)	972 (93.7)	**1,688 (88.2)**
Error with adverse health event**	160 (18.2)	65 (6.3)	**225 (11.8)**
Error with nonserious health event^††^	157 (17.9)	61 (5.9)	**218 (11.4)**
Error with serious health event	3 (0.3)	4 (0.4)	**7 (0.4)**
**Nonserious reports^§§,¶¶^**	**2,762 (94.3)**	**2,530 (96.8)**	**5,291 (95.5)**
Headache	343 (12.4)	285 (11.3)	**628 (11.9)**
Fatigue	318 (11.5)	257 (10.2)	**575 (10.9)**
Fever	299 (10.8)	262 (10.4)	**561 (10.6)**
Pain	293 (10.6)	231 (9.1)	**524 (9.9)**
Chills	254 (9.2)	205 (8.1)	**459 (8.7)**
Pain in extremity	209 (7.8)	167 (6.6)	**376 (7.1)**
Nausea	213 (7.7)	144 (5.7)	**357 (6.8)**
Dizziness	212 (7.7)	135 (5.3)	**347 (6.6)**
Injection site pain	138 (5.0)	121 (4.8)	**259 (4.9)**
COVID-19	169 (6.1)	89 (3.5)	**258 (4.9)**
Serious reports***^,†††^	**166 (5.7)**	**85 (3.3)**	**251 (4.5)**
Allergic reaction/Anaphylaxis	6	2	**8**
Appendicitis	4	1	**5**
Arrythmia	8	5	**13**
Atrial fibrillation	5	4	**9**
Atrioventricular node block, second or third degree	2	0	**2**
Supraventricular tachycardia	0	1	**1**
…Other	1	0	**1**
COVID-19	14	6	**20**
Death^§§§^	27	9	**36**
Dyspnea	4	1	**5**
Fall	1	6	**7**
Guillain-Barré syndrome	2	0	**2**
Hypertension, acute	7	3	**10**
Pericarditis^¶¶¶^	1	3	**4**
Pneumonia	6	1	**7**
Seizure	6	0	**6**
Thrombotic event	20	11	**31**
Stroke or transient ischemic attack	12	5	**17**
Pulmonary embolism	5	5	**10**
Other	3	1	**4**
Chest pain, not otherwise specified	9	3	**12**
Myocardial infarction	5	3	**8**
Myocarditis****	3	2	**5**

**Abbreviations**: MedDRA PT = Medical Dictionary for Regulatory Activities preferred term; VAERS = Vaccine Adverse Event Reporting System.

* Signs and symptoms in VAERS reports are assigned MedDRA PTs by VAERS staff members. Each VAERS report might be assigned more than one MedDRA PT, which can include normal diagnostic findings. A MedDRA PT does not indicate a medically confirmed diagnosis.

^†^ On August 31, 2022, the Food and Drug Administration authorized bivalent formulations of Moderna and Pfizer-BioNTech COVID-19 vaccines for use as a single booster dose ≥2 months after completing primary or booster vaccination, Pfizer-BioNTech for persons aged ≥12 years and Moderna for adults aged ≥18 years.

^§^ One report was for a person who received both Moderna and Pfizer-BioNTech bivalent booster doses at the same visit and did not experience an adverse health event.

^¶^ Vaccine administration or handling errors.

** The most common MedDRA PTs among reports of vaccination error included incorrect product formulation administered, incorrect dose administered, underdose, and wrong product administered.

^††^ Adverse health events coded for reports with nonserious vaccination errors included arthralgia, headache, injection site erythema, injection site swelling, fever, pain, and pain in extremity.

^§§^ Excluding vaccination error MedDRA PTs.

^¶¶^ Includes the top 10 most frequently coded MedDRA PTs among nonserious reports.

*** VAERS reports are classified as serious if any of the following are reported: hospitalization, prolongation of hospitalization, life-threatening illness, permanent disability, congenital anomaly or birth defect, or death. Serious reports to VAERS were reviewed by CDC physicians to form preliminary clinical impressions. https://www.meddra.org/how-to-use/basics/hierarchy

^†††^ Because of the small number of serious reports, percentages are not provided for serious report events. Other clinical impressions included acute pancreatitis, acute respiratory failure, aneurysm, arm pain, arthralgia, aseptic meningitis, bilateral pleural effusion, cellulitis, chronic anemia, compression fracture, confusion, contact dermatitis, costochondritis, erythema nodosum, fever, glaucoma, hearing loss, leukocytoplastic vasculitis, lower extremity weakness, lymphadenopathy, migraine, myalgia, pancreatitis, pericardial and pleural effusions, pericardial tamponade, pylephlebitis, rhabdomyolysis, unspecified bradycardia, unspecified tachycardia, transverse myelitis, vertigo, and vision loss.

^§§§^ For reports of death, cause of death was available for four reports: cardiac arrest, dementia, metastatic prostate cancer, and myocardial infarction.

^¶¶¶^ All four reports of pericarditis have been verified by medical record review.

**** Three of the five reports of myocarditis have been verified by medical record review.

Events related to vaccination errors (e.g., incorrect product formulation administered, incorrect dose administered, underdose, or wrong product administered) were commonly reported (1,913; 34.5%); among 877 reports of vaccination errors after receipt of Pfizer-BioNTech and 1,037 reports after receipt of Moderna bivalent booster doses, 225 (11.8%) reports indicated that an adverse health event had occurred.

Most VAERS reports (5,291; 95.5%) were classified as nonserious, including 2,762 (94.3%) after Pfizer-BioNTech and 2,530 (96.8%) after Moderna bivalent booster vaccination. The most commonly reported events among nonserious reports were headache (628; 11.9%), fatigue (575; 10.9%), fever (561; 10.6%), pain (524; 9.9%), and chills (459; 8.7%).

Among 251 VAERS reports classified as serious, five were reports of myocarditis, four were reports of pericarditis, and 20 were reports of COVID-19 disease. The age range of those who experienced myocarditis or pericarditis was 12–78 years and 46–78 years, respectively. Thirty-six deaths were reported; median age of decedents was 71 years (range = 46–98 years). For the four reports of death with sufficient information for review at the time of this report, cause of death included cardiac arrest, dementia, metastatic prostate cancer, and myocardial infarction. CDC has requested medical and vital records for the remaining decedents.

## Discussion

This report provides findings from v-safe and VAERS data collected during the first 7 weeks of bivalent Pfizer-BioNTech and Moderna mRNA booster dose administration among persons aged ≥12 years, when 22.6 million booster doses were administered in the United States. The findings in this report are generally consistent with those from safety data from preauthorization clinical trials of a BA.1 Omicron bivalent booster vaccination.****^,^^††††^

Reporting frequencies of reactions and health impacts among the 211,959 v-safe registrants aged ≥12 years who received an age-appropriate bivalent booster vaccination are similar to those described after receipt of first and second booster vaccine doses among adults aged ≥50 years (*3*–*5*). Among adults aged ≥18 years, reporting frequencies of local and systemic reactions after bivalent booster vaccination decreased with increasing age. This reporting pattern was also observed for primary series COVID-19 vaccination; v-safe registrants aged ≥65 years reported reactions less frequently after primary series doses than did younger adults (*6*).

Most reports to VAERS for persons aged ≥12 years after a bivalent booster dose were nonserious (95.5%) and were usually similar to those after first booster vaccination and second booster vaccination among adults aged ≥50 years (*3*–*5*). Vaccination errors were among the most common events reported to VAERS (34.5%); most (88.2%) of which did not list an adverse health event. Continued education of vaccine providers could help reduce administration errors.

Myocarditis and pericarditis are rare adverse events associated with receipt of COVID-19 mRNA vaccines (*2*). To date, five reports of myocarditis and four reports of pericarditis after bivalent booster vaccination were received by VAERS following administration of 22.6 million doses among persons aged ≥12 years in the United States. Reporting rates of myocarditis following COVID-19 mRNA primary series and monovalent booster vaccination were highest among adolescent and young adult males; myocarditis rates after monovalent booster dose in these early data are similar to or lower than those after primary series doses (*2*,*7*). In one study, an increased risk of pericarditis was detected in the first week after the second dose of COVID-19 mRNA vaccines among males aged 12–50 years and females aged 30–50 years (*8*).

Among nonserious reports to VAERS were 258 (4.9%) reports of COVID-19 disease; there were 20 (8.0%) serious reports of COVID-19 disease. Vaccine effectiveness studies have shown that among persons who were diagnosed with COVID-19, previous vaccination with mRNA-based vaccines reduced COVID-19 disease severity, including the risk of hospitalization and death (*9*,*10*).

The findings in this report are subject to at least three limitations. First, v-safe is a voluntary program; therefore, data might not be representative of the vaccinated population. Second, as a passive surveillance system, VAERS is subject to reporting biases and underreporting, especially of nonserious events (*1*). Finally, conclusions drawn from these data are limited by the 7-week surveillance period; safety monitoring will continue during the bivalent booster vaccination program.

As of October 12, 2022, ACIP recommends that all persons aged ≥5 years receive an age-appropriate bivalent mRNA booster dose ≥2 months after completion of a COVID-19 primary series or receipt of a monovalent booster dose (*3*). Preliminary safety findings after bivalent booster vaccination among persons aged ≥12 years are similar to those after monovalent booster vaccination (*3*–*5*). Health care providers and patients can be reassured that adverse events reported after a bivalent booster dose are consistent with those reported after monovalent doses. Health impacts after COVID-19 vaccination are less frequent and less severe than those associated with COVID-19 illness. CDC and FDA will continue to monitor vaccine safety and will provide updates as needed to help guide COVID-19 vaccination recommendations.

**Summary**

**What is already known about this topic?**

CDC recommended bivalent COVID-19 booster vaccination for persons aged ≥12 years in August 2022; approximately 22.6 million bivalent booster doses were administered during August 31–October 23, 2022.

**What is added by this report?**

Early safety findings from v-safe and the Vaccine Adverse Event Reporting System for bivalent booster doses administered to persons aged ≥12 years during the first 7 weeks of vaccine availability are similar to those previously described for monovalent vaccine booster vaccines.

**What are the implications for public health practice?**

Adverse events reported after a bivalent booster dose appear consistent with those reported after a monovalent booster and are less common and less serious than health impacts associated with COVID-19 illness.

## Footnotes

*
https://www.fda.gov/media/150386/download

†
https://www.fda.gov/media/144636/download

§
https://www.cdc.gov/vaccines/covid-19/clinical-considerations/interim-considerations-us.html

¶
https://vsafe.cdc.gov/en

**
https://vaers.hhs.gov

†† https://covid.cdc.gov/covid-data-tracker/#vaccination-demographic (Accessed October 23, 2022).

§§ Children and adolescents aged ≤15 years must be enrolled by a parent or guardian. Health check-ins are sent via text messages that link to web-based surveys on days 0–7 after vaccination; then weekly through 6 weeks after vaccination; and then 3, 6, and 12 months after vaccination.

¶¶ Under emergency use authorization regulations, health care providers are required to report certain adverse events after COVID-19 vaccination to VAERS, including death (https://vaers.hhs.gov/faq.html). VAERS forms ask for patient, vaccine, administration, and adverse event information. https://vaers.hhs.gov/docs/VAERS%202.0_Checklist.pdf

*** Each VAERS report might be assigned more than one MedDRA PT. A MedDRA-coded event does not indicate a medically confirmed diagnosis. https://www.meddra.org/how-to-use/basics/hierarchy

††† VAERS reports are classified as serious (based on FDA C.F.R. Title 21) if any of the following are reported: hospitalization, prolongation of hospitalization, life-threatening illness, permanent disability, congenital anomaly or birth defect, or death. https://www.accessdata.fda.gov/scripts/cdrh/cfdocs/cfcfr/cfrsearch.cfm?fr

§§§ 45 C.F.R. part 46, 21 C.F.R. part 56; 42 U.S.C. Sect. 241(d); 5 U.S.C. Sect. 552a; 44 U.S.C. Sect. 3501 et seq.

¶¶¶ Processed VAERS reports are those that have been coded using MedDRA, deduplicated, and undergone standard quality assurance and quality control review.

****
https://www.cdc.gov/vaccines/acip/meetings/downloads/slides-2022-09-01/07-COVID-Swanson-508.pdf

††††
https://www.cdc.gov/vaccines/acip/meetings/downloads/slides-2022-09-01/06-covid-miller-508.pdf
